# Tollip‐deficient zebrafish display no abnormalities in development, organ morphology or gene expression in response to lipopolysaccharide

**DOI:** 10.1002/2211-5463.13423

**Published:** 2022-06-27

**Authors:** Lidia Wolińska‐Nizioł, Karolina Romaniuk, Karolina Wojciechowska, Krzysztof Surga, Maciej Kamaszewski, Hubert Szudrowicz, Marta Miączyńska

**Affiliations:** ^1^ Laboratory of Cell Biology International Institute of Molecular and Cell Biology Warsaw Poland; ^2^ Zebrafish Core Facility International Institute of Molecular and Cell Biology Warsaw Poland; ^3^ Department of Ichthyology and Biotechnology in Aquaculture Institute of Animal Sciences University of Life Sciences Warsaw Poland

**Keywords:** CRISPR/Cas9, Gaucher disease, innate immunity, LPS, Tollip, zebrafish

## Abstract

Tollip is a multifunctional adaptor protein implicated in innate immunity, lysosomal trafficking/autophagy of protein aggregates and various signaling processes in mammalian models. To verify evolutionary conservation of these functions, we used CRISPR/Cas9 editing to construct a zebrafish line bearing a stable *tollip* knockout. In contrast to previously reported *tollip* morphants, Tollip‐deficient fish display normal development until adulthood, are fertile, and have no apparent physiological defects. When challenged with lipopolysaccharide (LPS), inflammatory gene expression is unaffected. Moreover, Tollip deficiency does not aggravate swimming deficiency resulting from lysosomal dysfunction and proteotoxicity in a fish model of Gaucher disease. Thus, individual functions of Tollip may be organism‐specific or manifest only upon certain conditions/challenges or disease backgrounds.

## Graphical abstract

Zebrafish bearing a stable knockout of *tollip* generated by genome editing display no developmental or physiological abnormalities and are fertile. Their inflammatory gene expression in response to lipopolysaccharide is unaltered. This is in contrast to a previous report on *tollip* knockdown phenotype by morpholinos (MOs) that was characterized by developmental defects and aberrant Wnt signaling.

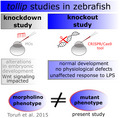

**Abbreviations**
dpf: days post fertilization
gDNA: genomic DNA
gRNA: guide RNA
HRM: high‐resolution melting
LPS: lipopolysaccharide
mpf: months post fertilization
PFA: paraformaldehyde
qPCR: quantitative real‐time PCR
TLR: Toll‐like receptors
Tollip: Toll‐interacting protein

## Introduction

Due to its well‐characterized biology and genetics the zebrafish (*Danio rerio*) is a prominent animal model to study gene function in the context of the whole organism and in various physiological processes [1]. Among them, a developing organism of zebrafish is suitable to investigate innate immune responses since the adaptive immune system including T‐ and B‐cell responses is not active until three weeks of development [2, 3, 4]. Among key inducers of innate immunity are Toll‐like receptors (TLR) that respond to external stimuli, including a constituent of the membrane of Gram‐negative bacteria, lipopolysaccharide (LPS) endotoxin [5]. Despite initial controversies about the machinery recognizing LPS in zebrafish [6, 7, 8], recent evidence clearly demonstrates that zebrafish possesses a *ly96* gene encoding Md‐2 and *tlr4*‐like genes whose products form the MD‐2‐TLR4 complex binding LPS, as in amniotes [9]. Although the mechanism of LPS action in zebrafish is not fully described, a general immune response to this endotoxin including transcriptomic modulation resembles the one observed in mammals [9, 10]. Thus, cellular mechanisms of TLR signaling can be addressed in zebrafish and a number of proteins acting in the TLR pathway have been already studied in this model, including Myd88 [11, 12], TRIF [13], TRAF6 [14], and Toll‐interacting protein, Tollip [15].

The role of an adaptor protein Tollip in the immune system has been initially characterized using *in vitro* models (reviewed in ref. [16]). Tollip was identified as an interactor of various immune receptors, including IL‐1R1, IL‐18R, and Toll‐like receptors TLR2 and TLR4 in mammalian cells [17, 18, 19], and more recently, as an interactor and stabilizer of STING [20]. Tollip was proposed to maintain immune cells in a quiescent state via binding and suppressing the activity of the interleukin‐1 receptor‐associated kinase 1 (IRAK‐1). Upon stimulation with IL‐1β or LPS, IRAK‐1 is phosphorylated and dissociates from the complex with Tollip to activate NF‐κB, AP‐1, and JNK inflammatory signaling [19]. Initial *in vivo* studies showed that Tollip knockout mice displayed no developmental abnormalities but reduced production of proinflammatory cytokines in response to sublethal doses of LPS [21]. However, the completeness of the used knockout has been questioned, due to a later discovery of multiple splicing isoforms of Tollip [22]. Moreover, no changes in production of proinflammatory cytokines were recently reported in the brain of Tollip knockout mice injected with LPS [23]. Thus, the exact contribution of Tollip to LPS response remains unresolved.

Furthermore, accumulating evidence indicates the involvement of Tollip in autophagic clearance of protein aggregates in cell culture models [16]. It was shown that Tollip acts as an autophagy adaptor by binding to ubiquitin (via its CUE domain) and to a key autophagy regulator Atg8/LC3. Both interactions are required for proper incorporation of huntingtin‐derived polyQ aggregates into the forming autophagosomes. Tollip was proposed to act cooperatively with another autophagy adaptor p62 (SQSTM1), which can also recognize and load cargo (such as misfolded ubiquitinated proteins) into autophagosomes for lysosomal degradation [24]. Cellular accumulation of misfolded proteins is characteristic for a growing list of ‘conformational diseases’, including neurodegenerative disorders such as Alzheimer’s, Parkinson’s, and Huntington’s disease, but also a broad range of other pathologies, ranging from lysosomal storage diseases, cancer to cystic fibrosis (reviewed by [25]). Moreover, a recent study described a regulatory role of Tollip in endosomal trafficking of damaged mitochondrial‐derived cargo to lysosomes via interaction with Parkin under mitochondrial stress conditions [26].

We have previously identified a function of Tollip as a negative regulator of the canonical Wnt pathway in mammalian cells [15]. We also investigated whether Tollip contributed to Wnt signaling during zebrafish development. The loss‐of‐function studies using morpholino antisense nucleotides demonstrated a number of developmental defects in *tollip* knockdown embryos. These developmental lesions pointed to the activation of canonical Wnt signaling and were rescued by downregulation of β‐catenin. Similarly, *tollip* overexpression caused morphological defects in the embryos suggesting a potential role of Tollip in the formation of body parts during development [15].

Considering a multitude of cellular roles of Tollip, such as those related to Wnt and TLR signaling, and lysosomal trafficking/autophagy, here we generated a stable Tollip‐deficient zebrafish line (*tollip^−/−^*). We wished to investigate the biological consequences of the lack of Tollip at an organism level and determine which of Tollip functions reported for mammalian models may be conserved also in fish. In detail, firstly, we wanted to verify our previous observations on a potential role of Tollip in developmental Wnt signaling [15]. Secondly, we wished to investigate the response of Tollip‐deficient embryos to LPS treatment. Thirdly, we decided to explore a possible contribution of Tollip to neurodegenerative diseases involving lysosomal impairment and protein aggregation, using a fish model of Gaucher disease [27].

## Results and Discussion

### CRISPR/Cas9 genome editing allows for successful generation of Tollip‐deficient zebrafish line

**Fig. 1:**
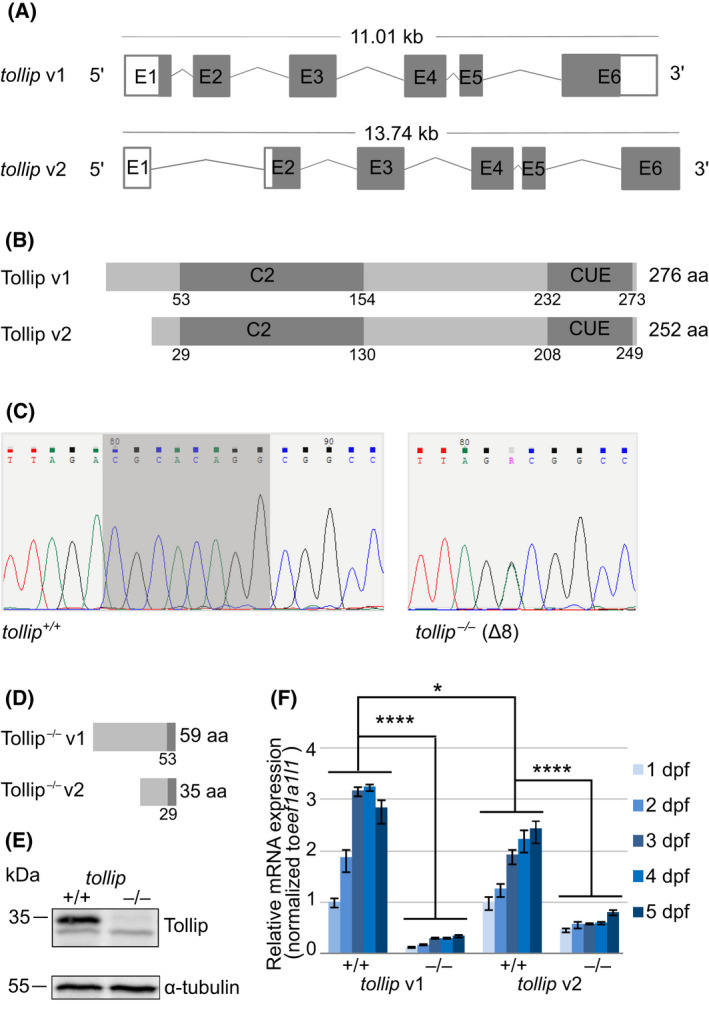
CRISPR/Cas9‐based genome editing allows for the generation of a Tollip‐deficient zebrafish line. (A) Schemes of the zebrafish *tollip* transcript variants 1 and 2 (v1 and v2), based on the Ensembl database, showing exons (E), translated sequences (gray), and UTR regions (white). (B) Schematic illustration of the structure of Tollip protein isoforms with the C2 and CUE domains indicated. (C) Partial DNA sequence of the target site within exon 2 of the *tollip* gene in wild‐type *tollip^+/+^* fish (left) and homozygous *tollip* ^−/−^ knockout fish (right). Deletion of eight nucleotides observed in the mutant line is shadowed in dark gray. There is an additional nucleotide change flanking the deletion (double peak marked R in the chromatogram, corresponding to A or G, with a predicted amino acid change D to G in the truncated protein product), indicating mosaicism of the generated line. (D) Schematic illustration of the predicted structure of Tollip protein isoforms synthesized from the mutated *tollip* gene. (E) Western blot of the 5 dpf protein lysates from the wild‐type (*tollip* ^+/+^) line and *tollip* ^−/−^ siblings. Top panel shows Tollip (~ 35 kDa) and a bottom panel shows α‐tubulin (~ 55 kDa) signal. (F) qPCR analysis of the expression of *tollip* transcripts during early zebrafish development (1–5 dpf). Bars represent the means ± SEM from 3–4 independent experiments (encompassing a pool of 10 larvae/condition). Mann–Whitney *U* test, *P < 0.05, ****P < 0.0001.

We used CRISPR/Cas9 genome editing to generate a stable zebrafish line carrying a premature stop codon within a relevant region of the *tollip* gene. In zebrafish there are two mRNA variants that give rise to two different isoforms of Tollip (Fig. 1A,B). To maximize the chance of ablating the protein function completely, we designed gRNA targeting the exon 2 whose sequence is common for both transcripts. Among the injected P0 fish we selected a founder fish harboring an 8 bp deletion in exon 2 (Fig. 1C). *In silico* analysis showed that the obtained genetic modifications, referred to as *tollip* ^−/−^, should result in a premature stop codon in the C2 domain (Fig. 1B,D). The F1 heterozygous progeny originating from the founder fish allowed obtaining F2 offspring, including the homozygous *tollip* ^−/−^ mutant line for further studies. Immunoblotting of total zebrafish embryo lysates (5 days post fertilization, dpf) using a mouse monoclonal antibody against Tollip confirmed the loss of full‐length protein in the homozygous *tollip* ^−/−^ line (Fig. 1E).

To investigate whether the observed lack of protein resulted from the reduced production of mRNA or regulation at the translational level, we analyzed the *tollip* mRNA expression profile in the first days of development (1–5 dpf). We have previously shown that *tollip* is highly expressed during early development of zebrafish [15]. The qPCR analysis showed that both mRNA variants were expressed in developing embryos, with the variant 1 (v1) being more abundant (Fig. 1F). Expression of both variants was significantly decreased in the mutant embryos (*tollip* ^−/−^) compared to wild‐type siblings (*tollip* ^+/+^) (Fig. 1F). The reduced levels of *tollip* mRNA likely result from nonsense‐mediated decay that eliminates mRNA molecules harboring premature termination codons to avoid the production of nonfunctional proteins [28].

### Tollip‐deficient zebrafish line bears no gross phenotypic alterations

**Fig. 2:**
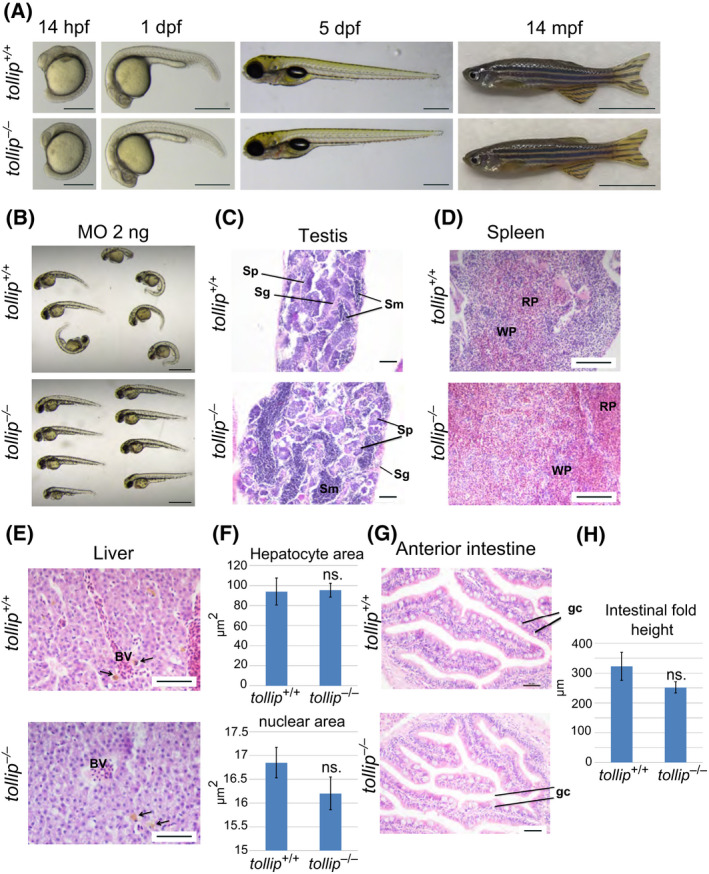
Knockout of the *tollip* gene has no effect on the fish morphology. (A) Wild‐type (*tollip* ^+/+^) and mutant (*tollip* ^−/−^) embryos at 14 h, and 1 and 5 days post fertilization (hpf, dpf) show normal development and morphology. Scale bar 500 μm. Right image, wild‐type (*tollip* ^+/+^) and mutant (*tollip* ^−/−^) male adults at 14 mpf are without visible body morphological defects. Scale bar 1 cm. (B) Morphology of wild‐type *tollip* ^+/+^ and mutant *tollip* ^−/−^ embryos injected with 2 ng of translation‐blocking morpholino targeting *tollip* (MO), observed at 48 hpf. Scale bar 1 mm. (C–H) Histology and quantitative analysis of hematoxylin and eosin stained sections of testes (C), spleen (D), liver (E, F), and anterior intestine (G, H) with specific cells and structures marked, including spermatozoa (Sm), spermatogonia (Sg), spermatocytes (Sp), red pulp (RP), white pulp (WP), blood vessels (BV), liver macrophages (arrows in E), and goblet cells (gc). For liver analyses, areas of hepatocytes and their nuclei are quantified in F. The height of intestinal folds is quantified in H. Scale bar 50 µm (C, G), 100 µm (D, E). Bars represent the means ± SEM (*n* = 3, each encompassing 40–60 (liver) or 25 (intestine) measurements from 5 selected areas) *t*‐test, ns.—nonsignificant.

We next analyzed the development of Tollip‐deficient embryos and observed no visible morphological alterations (Fig. 2A). These results are in contrast to our previous data when we used morpholinos to downregulate *tollip* expression in developing embryos [15]. In that study, *tollip* morphants exhibited the phenotypic hallmarks of abnormally activated Wnt signaling, such as pericardial edema, abnormal tail curvature, and inappropriate heart looping that were rescued by downregulation of β‐catenin [15]. These developmental aberrations were not reproduced here in genome‐edited fish bearing stable *tollip* knockout (Fig. 2A).

To clarify this discrepancy and assess possible off‐target effects of the previously used morpholino [15], we injected it into wild‐type and *tollip* ^−/−^ embryos. As shown in Fig. 2B, we observed that at a low‐dose, translation‐blocking morpholino targeting *tollip* induced developmental aberrations in wild‐type fish, as we previously reported [15]. Importantly, under the same conditions, *tollip* ^−/−^ knockout fish, which lack specific mRNA target for the morpholino, remained morphologically largely normal. These data suggest that the developmental defects observed in *tollip* morphants were specific and not primarily caused by off‐target effects of the morpholino. Instead, they could have resulted from acute depletion of Tollip protein at early stages of zebrafish development.

Numerous phenotypical discrepancies between knockdown morphants and knockout mutants were described in the literature, including also studies on Wnt‐related genes [29, 30]. In many cases such discrepancies are likely due to off‐target effects of morpholino nucleotides [31]. In some cases, however, long‐term genetic compensation in knockout mutants may mask phenotypes caused by acute, short‐term depletion of a given protein caused by morpholinos [32, 33]. Our data suggest that effects of the long‐term stable knockout of the *tollip* gene might be indeed compensated by other mechanisms, involving gene products unrelated to Tollip, as we did not find any sequences in the zebrafish genome with similarity to the *tollip* gene.

We further observed that Tollip‐deficient adults were fertile and morphologically indistinguishable from their wild‐type siblings (up to ~ 14 months post fertilization, mpf, Fig. 2A). Hematoxyline and eosine (H&E) staining of mutant testes showed a typical tissue pattern including seminiferous tubules in the parenchyma of testis (Fig. 2C). The morphology of the testicular tissue was not affected in *tollip* ^−/−^ siblings and the three major cell types, namely, spermatogonia, spermatocytes, and spermatozoa, were present. We further extended our histological analyses to compare the morphology of selected organs (spleen, liver, and intestine) and their resident immune cells in Tollip‐deficient and wild‐type adult fish. In parenchyma of the spleen, the red and white pulp were observed without any alteration within their structure (Fig. 2D). No aggregation of melanomacrophage centers was observed in the spleen tissue in *tollip* ^−/−^ compared to *tollip^+/+^* siblings, arguing that the immunological homeostasis was not altered in the mutant fish. In the liver of both genotypes, *tollip* ^+/+^ and *tollip* ^−/−^, hepatocytes had regular structure with a nucleus including one or more nucleoli (Fig. 2E). The distribution of hepatocytes was typical of the structure of the liver and their size parameters were unchanged (Fig. 2F). Similarly, in liver parenchyma of both genotypes, macrophages with normal distribution were observed. Analysis of cross sections of the intestine did not show any morphological differences of the *tollip* ^−/−^ organ compared to the *tollip* ^+/+^ genotype (Fig. 2G). Normal epithelial structure, including absorptive enterocytes and mucin‐producing goblet cells were observed. The intestinal fold height was not altered between the two genotypes (Fig. 2H). No inflammatory infiltrates or symptoms of disintegration of *lamina propria* were detected in the *tollip* ^−/−^ fish. To sum up, the microscopic analysis of the selected organs did not reveal any specific histological alterations in the *tollip* ^−/−^ line that could suggest any particular defects, for example, in the immune system.

### Tollip‐deficient larvae respond to LPS challenge with cytokine expression in a manner similar to wild‐type siblings

Although our histology analyses did not reveal any morphological alterations suggestive of impaired immune homeostasis, we investigated whether Tollip deficiency might lead to dysfunction of innate immune responses, as reported in mammalian models. To this end, we examined the response of Tollip‐deficient embryos to LPS.

**Fig. 3:**
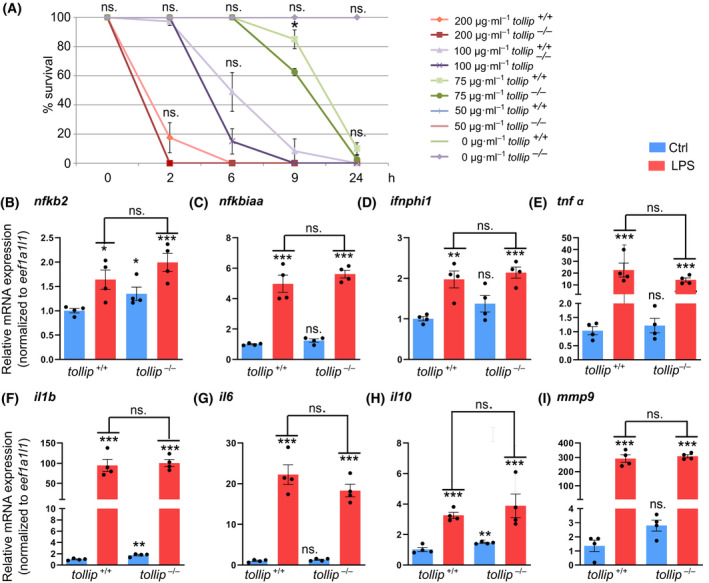
The expression of inflammatory mediators and regulators in response to LPS is largely normal in Tollip‐deficient larvae. (A) 24‐h LPS treatment induces the mortality in mutant and wild‐type larvae (3–4 dpf) in a dose‐dependent manner (an average of 4 experiments, 10 larvae per condition). (B–I) 6‐h exposure to LPS (75 µg·mL^−1^) induces expression of genes encoding NF‐κB components (*nfkb2*, *nfkbiaa*), interferon (*ifnphi1)*, proinflammatory cytokines (*tnfα*, *il1b*, *il6*), anti‐inflammatory cytokine (*il10*), and matrix metalloproteinase *mmp9* in wild‐type and *tollip^−/−^* 3 dpf larvae. Each dot represents data from one independent experiment (encompassing a pool of 10 larvae/condition), whereas bars represent the means ± SEM from *n* = 4 experiments. (A) Mann–Whitney *U* test, *P = 0.571; (B–I) ANOVA after log‐transforming the data, followed by Dunnett’s *post hoc* test, ns.—nonsignificant, *P < 0.01; **P < 0.005; ***P < 0.001.

We first tested different doses of LPS added to E3 medium to evaluate the potential differences in LPS‐induced mortality between *tollip* ^−/−^ and wild‐type control siblings. Concentrations of LPS below 50 µg·mL^−1^ did not cause any mortality among both groups (Fig. 3A). Although the differences in survival rates after exposure to moderate (75 µg·mL^−1^) and higher doses (100, 200 µg·mL^−1^) of LPS were in most cases not statistically significant, we noticed a tendency that mutant larvae died sooner (Fig. 3A). The highest (200 µg·mL^−1^) concentration of LPS applied for 2 h resulted in death of all mutant larvae (100%) and 82.5% in control wild‐type group. After 6‐h treatment with 100 µg·mL^−1^ LPS, the average survival rates of wild‐type and mutant larvae were 48.9% and 15%, respectively. A statistically significant difference in the survival rates between wild‐type and *tollip* ^−/−^ larvae (85% and 62.5%, respectively) was observed after 9‐h exposure to 75 µg·mL^−1^ LPS (*P* = 0.0571; Fig. 3A). Based on these results, for subsequent experiments measuring inflammatory gene expression we chose a LPS dose of 75 µg·mL^−1^ that when applied for 6 h had no effect on the survival, with mortality induced only at later time periods.

To assess the response to LPS at the transcriptional level, we measured expression of genes encoding mediators of NF‐κB activation and inflammatory cytokines, previously reported as targets of LPS‐induced signaling in zebrafish [12, 34] (Fig. 3B–I). We observed a statistically significant increase in basal, unstimulated expression of *nfkb2*, *il1b*, and *il10* in *tollip* ^−/−^ fish in comparison to the wild‐type fish (Fig. 3B,F,H). Similar trends, although statistically nonsignificant, were noted for the expression of *ifnphi1* and *mmp9* (Fig. 3D,I). In turn, basal expression of *nfkbiaa*, *tnfα*, and *il6* was at the same level in wild‐type and *tollip^−/−^* larvae (Fig. 3C,E,G). Altogether, these data suggest that the deficiency of Tollip may affect basal expression levels of some inflammatory mediators and cytokines in the developing organism that could imply a possible imbalance in the regulation of innate immunity responses.

As expected, LPS treatment strongly increased mRNA levels for all tested genes, encoding NF‐κB mediators and cytokines (Fig. 3B–I). However, we did not observe significant differences between *tollip^+/+^* and *tollip^−/−^* fish in response to LPS, arguing that Tollip deficiency in zebrafish does not affect gene expression in response to the endotoxin, at least at early stages of development. Thus, our results are in agreement with data on intranigral injections of LPS into mouse brains where no significant differences in cytokine gene expression were observed for wild‐type and Tollip‐deficient mice [23]. However, an earlier study reported reduced expression of inflammatory cytokines upon intravenous injection of LPS in Tollip knockout mice or LPS treatment of Tollip‐deficient macrophages [21]. It is therefore possible that Tollip may contribute to LPS response only in certain cell or tissue types, or at specific stages of organismal development.

### Tollip deficiency does not aggravate swimming deficiency of the *gba1* ^*−/−*^ mutant

Having verified that Tollip has no apparent role in innate immune response to LPS during early development of fish and considering reported roles of Tollip in vacuolar and lysosomal trafficking (reviewed in ref. [16]), we wished to investigate whether Tollip contributes to a pathology involving disturbed lysosomal and protein homeostasis, such as Gaucher disease. So far, a protective role of Tollip against protein aggregation was shown in human cells *in vitro* [24] but not yet investigated *in vivo* in animal models.

Gaucher disease is one of the most common lysosomal storage disorders, caused in humans by mutations in the *GBA1* gene encoding glucocerebrosidase. The resulting sphingolipid dysregulation leads to aberrant lysosomal degradation at the cellular level and clinically, to the dysfunction of multiple organs [35]. Studies of glucocerebrosidase‐deficient neurons showed that protein homeostasis was affected due to the accumulation of α‐synuclein oligomers [36] and impaired autophagy [37]. The zebrafish *gba1* ^−/−^ mutant displays many symptoms observed in humans, including progressive neurodegeneration and loss of motor activities by 12 weeks. At the molecular level, these fish exhibit an increase of lysosomal enzyme activity, mitochondrial dysfunction, increased levels of autophagic LC3‐II protein and extensive accumulation of ubiquitinated protein inclusions in the brain [27]. Therefore, the *gba1* ^−/−^ line represents an *in vivo* model of pathology involving lysosomal disorder and protein aggregation.

**Fig. 4:**
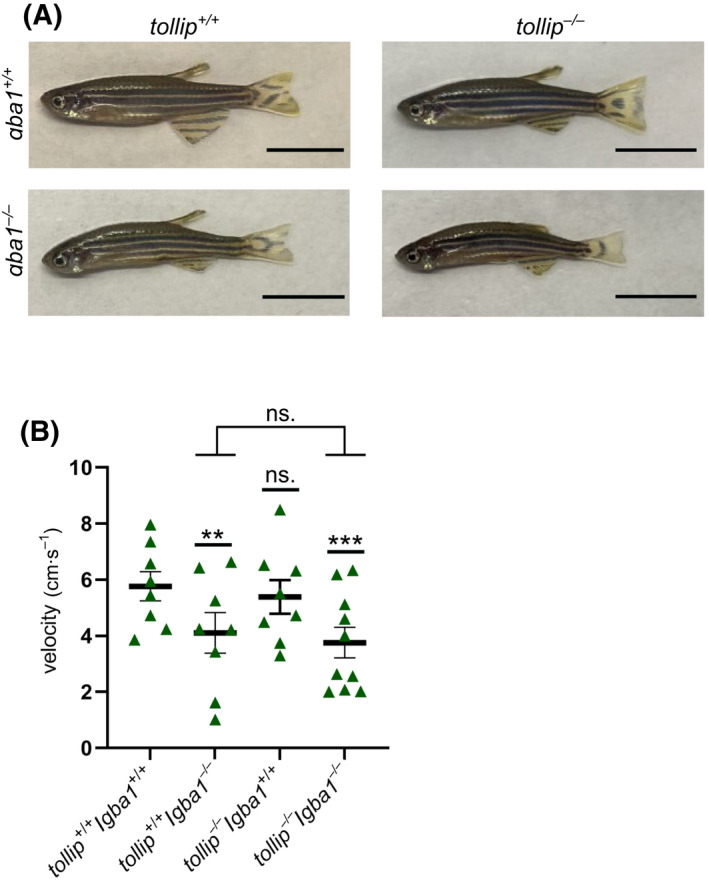
Deficiency of Tollip does not worsen the phenotypic hallmarks of Gaucher disease in the zebrafish model. (A) The general phenotype of zebrafish adults (3 mpf) of the examined mutant genotypes (*tollip* ^+/+^/*gba1* ^−/−^; *tollip* ^−/−^/*gba1* ^+/+^; *tollip* ^−/−^/*gba1* ^−/−^), compared to the wild‐type genotype (*tollip* ^+/+^/*gba1* ^+/+^). Scale bar 1 cm. (B) Swimming velocity examined in the mutant and wild‐type lines. Data are averaged within the tested groups, triangles indicate individual fish (*n* = 8 for the *tollip* ^+/+^/*gba1* ^+/+^, *tollip* ^+/+^/*gba1* ^−/−^, *tollip* ^−/−^/*gba*1^+/+/^ groups, *n* = 10 for the *tollip* ^−/−^/*gba1* ^−/−^ group) and the error bars represent SEM. *t*‐test, ns.—nonsignificant, **P < 0.005, ***P < 0.001.

To test whether Tollip deficiency would worsen the neurological phenotypes of the *gba1* ^−/−^ genotype, manifested by swimming abnormalities, we crossed *tollip* ^−/−^ fish with the *gba1* ^−/−^ line to obtain a double knockout mutant. Morphological analyses did not show any gross differences between the *gba1* ^−/−^ and the *tollip* ^−/−^/*gba1* ^−/−^ double mutant line at 3 mpf (Fig. 4A). As reported [27], the swimming activity of *gba1* ^−/−^ fish was significantly impaired compared to wild‐type individuals. In turn, the swimming velocity was not altered in the *tollip* ^−/−^ line compared to the wild‐type. Reduced swimming velocity was observed in *gba1* ^−/−^ and *tollip* ^−/−^/*gba1* ^−/−^ fish; however, no significant differences were detected between both tested lines (Fig. 4B). These results demonstrate that Tollip deficiency does not aggravate swimming deficiency in the fish model of Gaucher disease.

Overall, our study reports that stable knockout of the *tollip* gene in zebrafish does not impact embryonic development, body plan formation, normal adult physiology, or fertility. These data are in contrast to our previous study using morpholino‐mediated *tollip* knockdown in fish embryos [15] but are in agreement with similar observations on Tollip‐deficient mice [21]. Consistent with the proposed role of Tollip in the regulation of innate immunity responses in mammals [16], we noted that its deficiency may affect basal expression levels of some inflammatory mediators and cytokines in zebrafish embryos. Furthermore, although embryos lacking Tollip tended to survive worse upon LPS challenge, inflammatory gene expression induced in response to LPS was not affected that is consistent with some data in mammalian models [23]. Similarly, we did not detect measurable contribution of Tollip deficiency to the swimming deficiency observed in the *gba1* ^−/−^ Gaucher disease model fish, representing a progressive neurodegenerative pathology due to lysosomal disorder and proteotoxicity. Overall, this argues that individual functions of Tollip may not be universally conserved but rather organism‐specific or manifested only upon specific conditions, challenges/pathogens, or disease backgrounds.

## Materials and methods

### Zebrafish husbandry

Zebrafish (*Danio rerio*) mutant and wild‐type lines (AB, ABTL) were housed in a recirculating aquaria system at 28.0 °C in the Zebrafish Core Facility (International Institute of Molecular and Cell Biology in Warsaw, IIMCB). Embryos and larvae were raised at 28.5 °C in E3 medium and staged as described [38]. Procedures concerning maintenance of fish, handling, spawning and tissue collection (0–5 dpf) were conducted in accordance with the animal welfare guidelines operating at the IIMCB based on Polish Act on the Protection of Animals Used for Scientific or Educational Purposes of January 15, 2015. Generation, maintenance, and use of mutant lines were approved by the Ministry of Environment (Decision No. 180/2017). Experiments on adult fish were conducted according to the protocol approved by the Second Local Commission for the Ethics of Animal Experimentation in Warsaw (permits no. WAW2/058/2019, WAW2/022/2021).

### Zebrafish lines

The *tollip* ^−/−^ line was generated using the CRISPR/Cas9 system in the ABTL background. The mutant *gba1^c.1276_129del^* zebrafish line, here denoted as *gba1* ^−/−^, was previously described [27] and obtained from Prof. Oliver Bandmann (University of Sheffield).

### Generation of Tollip‐deficient zebrafish line using the CRISPR/Cas9 method

To identify specific sequences for CRISPR/Cas9‐directed mutagenesis, the CHOP (http://chopchop.cbu.uib.no/) web tool was used. The target site: 5′‐AGTGCAGTTAGACGCACAGGCGG‐3′ was selected within exon 2 of the *tollip* gene. Template for gRNA *in vitro* transcription was prepared via PCR under standard thermal conditions using primers listed in Table 1. The final PCR fragment was purified with QIAquick PCR purification kit (Qiagen, Hilden, Germany, #28106) and transcribed using MEGAshortscript T7 transcription kit (Life Technologies, Carlsbad, CA, USA, #AM1354). Generated gRNA was purified by miRNeasy Mini kit (Qiagen, #217004). The Cas9 RNA was synthesized from pCS2‐nCas9n (a gift from Wenbiao Chen, Addgene plasmid, #47929 [39]) using mMESSAGE mMACHINE T7 Transcription Kit (Life Technologies, #AM1344M).

**Table 1:** List of primers used in the study.

Gene	Primer name	Method/purpose	Sequence 5' → 3'
*tollip*	tollip_gRNA_ex2_F	Guide RNA synthesis targeting exon 2	GCGTAATACGACTCACTATAGAGTGCAGTTAGACGCACAGGGTTTTAGAGCTAGAAATAGCAAGTTAAAATAAGGCTAGTC
	tollip_gRNA_constant_R		GATCCGCACCGACTCGGTGCCACTTTTTCAAGTTGATAACGGACTAGCCTTATTTTAACTTGCTATTTCTAGCTCTAAAAC
*tollip (ex2)*	F	HRM analysis	TTCTGAGGATCATGCCGACT
	R		CGTCCAGCAGTGCCTAAAGA
*tollip*	F	Sequencing	GTCATGAAGCACATCACACCAA
	R		ACAGTGTCTTGGAGTGGGTT
*tollip v1*	F	qPCR	GGTTTTGAGCTCCGCTGATG
	R		TGTAAACCTGTCCTCGTTGC
*tollip v2*	F		GTCGAGCTCCTTGCTGTCTA
	R		TTGCAAGCTTAGCCTGTACT
*nfkb2*	F		TGGCTGGAGCACTAAGGATG
	R		CCTCTCTGCTTTGGCTCCTC
*ifnphi1*	F		CTGACCTCAAAGAATGTGTGGC
	R		CTTGCGTTGCTTGCGATGAT
*nfkbiaa*	F		ACAACCGAAGAGAGAACATGGA
	R		CGAAATCTCCCGCGTCTCAT
*tnfa*	F		CGCTGGTGATGGTGTCTAGG
	R		CCCTGGGTCTTATGGAGCGT
*il1b*	F		CGTACTCAAGGAGATCAGCGG
	R		GCGGTGCTGATAAACCAACC
*il6*	F		GGCATTTGAAGGGGTCAGGA
	R		GCGTTAGACATCTTTCCGTGC
*il10*	F		GACCATTCTGCCAACAGCTC
	R		ACCCCCTTTTCCTTCATCTTTTC
*mmp9*	F		CGGGAACAGCAATGAAGCAC
	R		GCCGTATCTCTGTTAGGGCAG
*elfa1a1L1*	F		TCCTCTTGGTCGCTTTGCTG
	R		GTGTGATTGAGGGAAATTCACTTG

Fertilized eggs were obtained from natural spawning of pair‐wised adult zebrafish. One‐cell ABTL zebrafish embryos (70–100) were injected with 1 nL of mixture of Cas9 mRNA (300 ng·μL^−1^) and gRNA (100–300 ng·μL^−1^) containing 0.05% phenol red. Injected embryos at 2 dpf were screened for introduced genetic alterations within the P0 generation including potential founders using standard PCR and sequencing procedures. To test this, genomic DNA (gDNA) was extracted from 10 embryos. Briefly, anesthetized (in ice water) individual embryos were suspended in 20 μL of TE buffer pH 8.0 (1 m Tris, 0.5 m EDTA) and incubated at 95 °C for 10 min. Next, 0.5 μL of Proteinase K (20 mg·mL^−1^; EURx, Gdańsk, Poland, #E4350) was added to the sample and further incubation at 55 °C for 60 min was performed followed by inactivation of the enzyme at 95 °C for 10 min and centrifugation (10 000 ***g*** for 1 min). At 5 dpf, the remaining embryos from the injected group were transferred to the aquaria system where they were grown to adulthood. Three‐month‐old fish (P0) were individually outcrossed to screen the progeny (F1, 10–12 embryos, 2 dpf) for the germline transmission and to identify the indel mutations based on the sequencing results. The remaining offspring (F1) of the identified founder (P0) was maintained in the system to reach the sexual maturity. In case of genotyping the adult generations, fish were anesthetized in tricaine methanesulfonate and gDNA was isolated from a small piece of tail fin suspended in 50 μL of TE buffer following the digestion with Proteinase K at 55 °C for 1.5 h and its inactivation. 1–2 μL of the supernatant of purified gDNA was used as a template for standard PCR or high‐resolution melting (HRM) analysis.

### Standard PCR and sequencing

Primers were designed using Primer3 software (http://biotools.umassmed.edu/bioapps/primer3_www.cgi; Table 1). The PCR was made with 2 μL of 10× PCR buffer, 2 μL of dNTP (2.5 mm), 1 μL of each primer (10 μm), 0.3 μL of Taq DNA polymerase (Qiagen, #2012015), 2 μL of genomic DNA, and water up to 20 μL. The PCR thermal protocol was 94 °C for 3 min, then 35 cycles of 94 °C for 1 min, 57 °C for 30 s and 72 °C for 45 s, and finally 72 °C for 10 min. Samples were sequenced using Sanger method.

### High‐resolution melting (HRM) analysis

Primers were designed using Primer3 software (Table 1). The PCRs contained 5 μL of the RT PCR Mix EvaGreen^®^ (AA Biotechnology, Gdańsk, Poland, #2008‐100G), 4 μL of primer mixture (0.7 μm of each primer), 1 μL of gDNA, and water up to 10 μL. The PCR was performed in the LightCycler 480 instrument (Roche, Basel, Switzerland). The thermal profile of two‐step PCR was 95 °C for 7 min, then 40 cycles of 95 °C for 10 s and 60 °C for 20 s, followed by 95 °C for 15 s and 60 °C for 15 s and the final increasing the temperature by 0.07 °C·s^−1^ to 95 °C. Curves were analyzed using the lightcycler sw 1.1 software system (Roche).

### qPCR

The total RNA was isolated from 10 pooled embryos/larvae at the indicated time points using Total RNA Mini Kit (AA Biotechnology, #031‐25). Purified RNA was eluted with RNase‐free water to a final volume of 50 μL. Quality and quantity of RNA samples were assessed using the Nanodrop 2000c spectrophotometer (Thermo Fisher Scientific, Waltham, MA, USA). Then, 500 ng of RNA was treated with DNase (Sigma‐Aldrich, St. Louis, USA, #AMPD1‐1KT) followed by cDNA synthesis using RevertAid First Strand cDNA Synthesis Kit (Thermo Fisher Scientific, #K1622) according to the manufacturer’s instructions. qPCR primers (Table 1) for *tollip* mRNA transcripts and immunological marker genes were designed using the Primer‐BLAST web tool to target the exon‐exon junctions and thus to increase their specificity by avoiding amplification of gDNA. Two sets of primers were designed to determine the expression level of two existing transcript variants of the *tollip* gene. Each well of PCR plate contained 1 µL of cDNA as a template, 0.5 µm of each primer, and 5 µL KAPA SYBR FAST qPCR Master Mix (Kapa Biosystems, Wilmington, MA, USA,  #KK4618) and water up to 10 μl. The PCR was performed in the Fast Real‐Time PCR thermocycler (Applied Biosystems) under standard thermal conditions: 95 °C for 10 min, then 40 cycles of 95 °C for 15 s and 60 °C for 1 min followed by the melting curve analysis. *elfa1a1L1* was chosen as a reference gene. qPCR data were analyzed by the ΔΔ*C*_t_ method and presented as fold changes. The results are representative of 3–4 biological replicates that were performed in technical duplicates.

### Morpholino injections

Morpholino injections were performed as previously described [15]. In brief, translation‐blocking morpholino oligonucleotide targeting *tollip* (ATG2 5′‐CTGCTGCTGAGTCGGCATGATCCTC‐3′) was obtained from Gene Tool (Philomath, OR, USA). 2 ng morpholino was microinjected into the yolk of 1‐cell stage of *tollip* ^+/+^ and *tollip* ^−/−^ embryos to observe the phenotypic effects at 2 dpf.

### SDS/PAGE and western blot analysis

Proteins were extracted from 5 pooled larvae (5 dpf) rinsed three times with cold Ringer’s buffer supplemented with protease inhibitors (6 μg·mL^−1^ chymostatin, 0.5 μg·mL^−1^ leupeptin, 10 μg·mL^−1^ antipain, 2 μg·mL^−1^ aprotinin, 0.7 μg·mL^−1^ pepstatin A, and 10 μg·mL^−1^ 4‐amidinophenylmethanesulfonyl fluoride hydrochloride, 1 mm EDTA), dissolved in 5 µL 1× Laemmli buffer per embryo and then lysed by sonication for 20 s. After incubation at 95 °C for 5 min and centrifugation for 10 min (10 000 g), samples were separated using 15% SDS/PAGE and transferred to nitrocellulose membrane (Whatman, Maidstone, Kent, UK) using Mini‐PROTEAN Tetra Cell System (Bio‐Rad, Hercules, CA, USA). Then, the membrane was blocked in 5% skim milk for 1 h and probed with specific primary antibodies (mouse anti‐Tollip, R&D Systems, Minneapolis, MN, USA, #MAB4678; mouse anti‐α‐tubulin, Sigma‐Aldrich, #T5168) overnight at 4 °C followed by incubation at room temperature for 1 h with secondary horseradish peroxidase (HRP)‐conjugated antibodies (anti‐mouse IgG #111‐035‐062 and anti‐rabbit #111‐035‐144, Jackson ImmunoResearch, West Grove, PA, USA). Then, membranes were incubated with Clarity Western ECL Substrate (Bio‐Rad, #170‐5061) and imaged using the ChemiDoc MP system (Bio‐Rad). α‐tubulin was used as a loading control.

### Microscopy

For live imaging of zebrafish wild‐type and mutant embryos during early development, the Leica M60 microscope (Leica Microsystems, Wetzlar, Germany) with Leica DMC2900 camera was used. Pictures were acquired using leica application suite V4.3.0 software (Leica Microsystems). Whole‐body images of adult fish were taken with a commercial digital camera.

### Histological analyses

Zebrafish at 3 mpf were anesthetized and fixed in 4% paraformaldehyde (PFA) at room temperature. To maximize the penetration of PFA to internal organs, individuals were incised ventrally midline from the anal pore to the base of the pectoral fin. Fixation was followed by the dehydration procedure using ethanol solutions of increasing concentrations (from 50 up to 99.8%). Solutions were changed in two‐day intervals. Then, samples were cleared in xylene, embedded in paraffin, and cut into 5‐μm‐thick sections using a Leica RM2025 microtome (Leica Microsystems). The obtained cross sections were stained with hematoxylin–eosin (H&E). Histomorphological analyses of the slides were conducted, measuring the area of hepatocytes and their nuclei (for liver sections) and the height of intestinal folds (for anterior intestine sections). Microscopic observations of liver, spleen, anterior intestine, and testes were carried out using the Nikon Eclipse 90i microscope with Nikon Digital Sight DS‐U1 camera (Nikon Corporation, Tokyo, Japan). Images were acquired using the nis‐elements ar 2.10 software (Nikon Corporation).

### LPS treatment

Larvae (*tollip* ^+/+^ and *tollip* ^−/−^, 3 dpf) were exposed to different LPS (Sigma‐Aldrich, #L439) doses (25, 50, 75, 100, 200 µg·mL^−1^) dissolved in E3 embryo medium for 24 h according to the previously reported procedure [34]. Briefly, 3 dpf zebrafish were exposed to LPS by static immersion at 28.5 °C. Dead larvae were counted at 2, 6, 9, and 24 h after LPS in addition to calculate the survival rates for each LPS concentration.

### Behavioral analysis

Fish had been moved from husbandry tanks to the behavioral room 1 day before the analysis was performed and were not fed during that time. Movements were recorded for 15 min following 30‐min acclimation time in ZebraCube cabinet (ViewPoint, Lyon, France). Camera positioned at the top was used to track the movements of individual zebrafish. Signals were gathered from the side using mirror system and from the top. Locomotion activity of fish (swimming velocity) was quantified using viewpoint zebralab v. 3.12 analysis software. Signals above 40 cm·s^−1^ were cut off as outliers.

### Data analysis and statistics

Graphs and statistical analysis were performed using Microsoft Excel and graphpad prism version 8.4.2 (GraphPad Software, San Diego, CA, USA). For all datasets, tests for normality were performed followed by the appropriate statistical test (*t*‐test, one‐way analysis of variance (ANOVA) followed by Dunnett’s *post hoc* test, or Mann–Whitney *U* test) and the null hypothesis was rejected at a *P*‐value of 0.05 or lower.

## Conflict of interest

The authors declare no conflict of interest.

### Author contributions

LWN conceived the research, designed, and analyzed the experimental work with support from MM. LWN and KR performed the majority of the experiments with support from KW (searching for founder fish, optimization of methods), KS (behavioral experiments), and MK and HS (histological experiments and analysis the results). LWN and MM wrote the manuscript.

## Data Availability

The data that support the findings of this study are available from the corresponding author miaczynska@iimcb.gov.pl upon reasonable request.
